# Gene–Environment Interactions in Severe Mental Illness

**DOI:** 10.3389/fpsyt.2014.00048

**Published:** 2014-05-15

**Authors:** Rudolf Uher

**Affiliations:** ^1^Department of Psychiatry, Dalhousie University, Halifax, NS, Canada; ^2^Department of Psychology and Neuroscience, Dalhousie University, Halifax, NS, Canada; ^3^Social, Genetic and Developmental Psychiatry Centre, Institute of Psychiatry, King’s College London, London, UK

**Keywords:** gene–environment interactions, genome-wide association studies, schizophrenia, bipolar disorder, major depressive disorder, severe mental illness

## Abstract

Severe mental illness (SMI) is a broad category that includes schizophrenia, bipolar disorder, and severe depression. Both genetic disposition and environmental exposures play important roles in the development of SMI. Multiple lines of evidence suggest that the roles of genetic and environmental factors depend on each other. Gene–environment interactions may underlie the paradox of strong environmental factors for highly heritable disorders, the low estimates of shared environmental influences in twin studies of SMI, and the heritability gap between twin and molecular heritability estimates. Sons and daughters of parents with SMI are more vulnerable to the effects of prenatal and postnatal environmental exposures, suggesting that the expression of genetic liability depends on environment. In the last decade, gene–environment interactions involving specific molecular variants in candidate genes have been identified. Replicated findings include an interaction between a polymorphism in the *AKT1* gene and cannabis use in the development of psychosis and an interaction between the length polymorphism of the serotonin transporter gene and childhood maltreatment in the development of persistent depressive disorder. Bipolar disorder has been underinvestigated, with only a single study showing an interaction between a functional polymorphism in the *BDNF* gene and stressful life events triggering bipolar depressive episodes. The first systematic search for gene–environment interactions has found that a polymorphism in *CTNNA3* may sensitize the developing brain to the pathogenic effect of cytomegalovirus *in utero*, leading to schizophrenia in adulthood. Strategies for genome-wide investigations will likely include coordination between epidemiological and genetic research efforts, systematic assessment of multiple environmental factors in large samples, and prioritization of genetic variants.

## Severe Mental Illness

Severe mental illness (SMI) includes the most disabling psychiatric disorders that typically require inpatient treatment, such as schizophrenia, bipolar disorder, and severe depression. Family and molecular genetic studies suggest that schizophrenia, bipolar disorder, and major depressive disorder share common etiology and there may be advantages in studying these disorders jointly (1–4). This review focuses on these three disorders. Studies of subthreshold psychotic and mood symptoms are also included since they may provide additional information on etiology of SMI.

Both genetic disposition and environmental exposures play important roles in the development of SMI. The risk of SMI runs in families and is shared in proportion to the degree of biological relatedness (5, 6). The overall contribution of genetic factors appears to be stronger for SMI than for common mental disorders (6). Twin studies consistently estimate the heritability of schizophrenia and bipolar disorder in the range of 70–80% (7–9). The genetic contribution to depression may depend on severity: while general population-based studies find a relatively low heritability around 38% (10), the heritability of hospital-ascertained severe depression was estimated to be between 48 and 75% (11). Molecular genetic studies have recently identified a number of specific genetic polymorphisms that directly contribute to schizophrenia, bipolar disorder, or all types of SMI across populations (12–14). The majority of the genetic variants may confer risk to more than one type of mental illness (1, 12).

A number of environmental factors contribute to SMI (Table 1). *In utero* exposure to infection, lack of nutrients, maternal stress, perinatal complications, social disadvantage, urban upbringing, ethnic minority status, childhood maltreatment, bullying, traumatic events, and cannabis use have all been found to contribute to one or more types of SMI. Some of these exposures appear to be responsible for substantial proportion of cases of SMI. For example, the availability of vitamin D during the prenatal development may be responsible for 44% cases of schizophrenia (15), childhood maltreatment and bullying account for 33% of cases of schizophrenia (16), urban birth and upbringing may be responsible for 35% of cases (17), and use of cannabis in adolescence may account for 14% of cases of schizophrenia (18). A quick addition shows that the above attributable risk percentages sum up to more than 100%. This suggests that multiple factors are likely to contribute to each case of schizophrenia. Some risk factors may be correlated (e.g., a child growing up in urban setting may be more likely to be maltreated) or they may act in synergy (e.g., a person whose early brain development was affected by a lack of vitamin D may be less resilient to the effects of cannabis in adolescence). Nonetheless, the high attributable risks strongly suggest that a significant proportion of cases of SMI may be preventable through modification of environment.

**Table 1:** **Environmental risk factors for severe mental illness**.

	Exposure	Schizophrenia	Bipolar disorder	Major depressive disorder
Prenatal	Season of birth	+++ (17)	++ (19)	+ (19)
	Inadequate nutrition	++ (20)	++ (21)	+ (21)
	Vitamin D levels	+++ (15)		
	Lead	+ (22)		
	Herpes simplex virus-2	++ (23)		
	Rubella	+ (24)		
	Prenatal stress		+ (25)	+ (25)
Perinatal	Preterm birth	++ (26)	+++ (26)	+ (26)
	Obstetric complications	+ (27)	−(28)	
	Hypoxia			
Childhood	Cytomegalovirus	+ (29)		
	Maltreatment	+++ (16)	+ (30–32)	+++ (33, 34)
	Loss of a parent			++ (35)
	Social disadvantage	+++ (36, 37)	−(36)	+++ (36, 38)
	Bullying	++ (39)		+ (40)
	Urbanicity	+++ (71)		
	Minority status	+++ (41)		++ (42)
Adolescence	Cannabis	+++ (18)	+ (30)	+ (18)
Adulthood	Stressful life events	+ (43)	++ (44)	+++ (45)
	*Toxoplasma*		+ (46)	

*The number of plus signs indicates the strength of evidence for association: +++, consistent evidence from multiple studies or a meta-analysis; ++, evidence from several studies or a strong association in a high-quality study; +, evidence from a single study or multiple low quality studies; −, evidence for no association; blank fields reflect lack of evidence for or against association. The list is limited to environmental factors and excludes risk factors that reflect condition of the individual (e.g., birth weight)*.

## Gene–Environment Interactions

Gene–environment interactions reflect a causal mechanism where one or more genetic variants and one or more environmental factors contribute to the causation of a condition in the same individual with the genetic factors influencing the *sensitivity* to environmental exposures (47, 48). They should be distinguished from gene–environment correlations, where genetic factors influence the *probability* of environmental exposures. Statistically, the likelihood of a gene–environment interaction being present is usually inferred from a significant interaction term between genetic and environmental factor in a multiple regression. Since statistical inference and power depend on the distribution of both the environmental factor and the genetic variant in a particular sample, statistical results often do not correspond to actual biological interaction (49, 50). Therefore, multiple methods of inquiry are required to establish whether a gene–environment interaction is involved (51).

**Figure 1:**
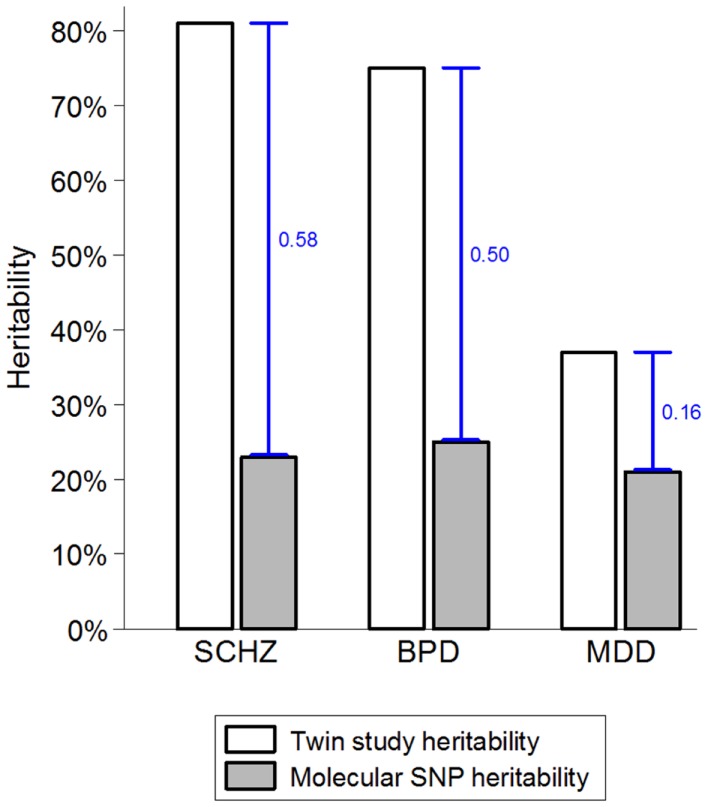
**The heritability gap**. Heritability estimates from twin and molecular genetic studies for schizophrenia (SCHZ), bipolar disorder (BPD), and major depressive disorder (MDD) are based on review of twin studies and the results from the Cross-disorder Group of the Psychiatric Genetic Consortium (1, 55). Heritability gap is marked by a blue capped line and quantified as the proportion of total variance in the presence of each disorder. Possible explanations for the heritability gap include gene–environment interactions, inherited rare genetic variants, and overestimation of heritability in twin studies.

While there are strong environmental risk factors that contribute to a large proportion of cases of SMI, there is also significant evidence of resilience and major individual differences in the impact of environmental exposures (52, 53). Several strong indicators suggest that the marked individual differences in sensitivity to potentially pathogenic exposures are, at least partially, due to genetic factors (54, 55). The combination of very high heritability and strong environmental factors suggests that a large proportion of cases of SMI must be due to a synergy between genetic and environmental causes. If a single environmental factor can explain 30 or 40% of cases of a disease that is 80% heritable, then some of the heritability must be due to joint causation by genes and environment. The way heritability is estimated in twin studies means that gene–environment interactions involving environmental factors that are shared within a family are attributed to the genetic component and contribute to heritability estimates (55–57). This misattribution of gene–environment mechanisms to heritability may account for two ostensibly paradoxical observations. First, while some of the strongest known environmental factors (e.g., urbanicity and social disadvantage) are shared within families, twin studies typically estimate no or very small contribution of shared environment (58–60). Second, since it has recently become possible to quantify the genetic contribution using molecular genetic data, it became apparent that genetic variants account for much smaller proportion of variance than the twin-based heritability estimates suggested (Figure 1). One of the most likely explanations for the heritability gap is that gene–environment interactions involving shared environmental factors are part of the twin heritability estimates but do not contribute to the molecular heritability estimates that are based on unrelated individuals (4, 55). The large “heritability gaps” for schizophrenia and bipolar disorder suggest that gene–environment interactions may potentially explain a large proportion of cases of SMI.

## Gene–Environment Interactions by Proxy

Several studies have attempted to estimate gene–environment interactions using the familial loading of risk for mental illness as a proxy for genetic factors. A Finnish study has shown that family history of schizophrenia interacts with low birth weight in their effect on educational achievement (61). The link between low birth weight and low educational achievement was much stronger among offspring of biological parents with schizophrenia than in children with no family history of SMI. Since low educational achievement is an antecedent to schizophrenia and major depressive disorder (62), this study may be interpreted as suggesting that gene–environment interactions operate in the early processes on the neurodevelopmental pathway to SMI. This interpretation depends on the assumption that low birth weight is a reflection of environmental factors during pregnancy. However, since a genetic contribution to birth weight is likely (63), the interpretation may become more complex. Several other studies have explored similar proxy gene–environment interactions leading to schizophrenia and other psychotic disorders. A longitudinal Finnish adoption study has shown that excellent parenting and clear communication can substantially reduce the risk of schizophrenia and related conditions among adopted offspring of biological mothers with schizophrenia while no effect of parenting was seen in adopted offspring of biological mothers without SMI (64). Another Finnish study derived data from a population-based registry and showed that serious infection during pregnancy increased the risk of psychosis in offspring who had a family history of psychotic illness (65). A large-scale Swedish adoption study has shown that socio-economic disadvantage during upbringing increased the risk of psychosis in adoptees with a family history of SMI in biological relatives (66). Yet, the pattern is not uniform: a recent study has found a correlation between family history of psychosis and childhood maltreatment (with sons and daughters of parents with psychosis being more often maltreated by their parents), but no interaction between family history of psychosis and childhood maltreatment in the causation of psychotic disorders (67). A twin study of depression found that genetic disposition, indexed by depression in monozygotic and dizygotic co-twins, significantly interacted with environmental triggers (stressful life events) in leading to depressive episodes (68). Taken together, these studies show that pathogenic effects of many but not all environmental risk factors depend on the familial disposition to SMI. Since several of the studies were adoption or twin studies, the familial disposition was separated from the environmental factors and it can be interpreted as a proxy of genetic effects. However, even in adoption studies, there is a residual sharing of environment in the early life and in twin studies monozygotic twins may share more of their environment than dizygotic twins. Consequently, the interpretation of gene–environment studies using proxy measures is limited because familial relatedness cannot be equaled to genetic contribution and because specific environmental factors may interact with specific genetic variants rather than with the multitude of risk alleles that may constitute familial disposition. Therefore, investigation of gene–environment interactions involving specific molecular genetic variants is necessary to advance our knowledge of causal mechanisms leading to SMI.

## Gene–Environment Interactions Involving Specific Molecular Genetic Variants

Molecular genetic variants can be measured with high accuracy and their identification may help the development or novel indications for therapeutics. Gene–environment interactions with specific molecular genetic variants have started to be identified in the last decade. Most of the findings have concerned community-ascertained depression or other relatively common mental disorders (69). More recently, several groups of researchers have also investigated and identified specific gene–environment interactions that play a role in the causation of schizophrenia and related conditions (Table 2).

**Table 2:** **Molecular gene–environment interactions in severe mental illness**.

Gene	Exposure	Outcome	Original report	Replication	
	
			Reference	Result	Reference
*BDNF*	Stressful life events	Depression	Kim et al. (70)	Yes	Brown et al. (71)
*CRHR1*	Childhood maltreatment	Depression	Bradley et al. (72)	Yes	Polanczyk et al. (73)
*HTR2A*	Parenting	Depression	Jokela et al. (74)		
*MAOA*	Childhood adversity	Depression	Cicchetti et al. (75)	Yes	Melas et al. (76)
*NR3C1*	Childhood adversity	Depression	Bet et al. (77)		
*SLC6A4*	Childhood maltreatment	Depression	Caspi et al. (78)	Yes	Karg (79)
*SLC6A4*	Stressful life events	Depression	Caspi et al. (78)	Y/N	Karg et al. (79), Brown et al. (80)
*BDNF*	Stressful life events	Bipolar depressive episodes	Hosang et al. (81)		
*COMT*	Cannabis	Schizophrenia/psychosis	Caspi et al. (82)	No	Zammit et al. (83)
*COMT*	Cannabis + childhood maltreatment	Schizophrenia/psychosis	Alemany et al. (84)	Yes	Vinkers et al. (85)
*AKT1*	Cannabis	Schizophrenia/psychosis	van Winkel and Genetic Risk and Outcome of Psychosis (GROUP) Investigators (86)	Yes	di Forti et al. (87)
*BDNF*	Childhood maltreatment	Schizophrenia/psychosis	Alemany et al. (88)	No	Ramsay et al. (89)
*FKBP5*	Childhood maltreatment	Psychotic symptoms	Collip et al. (90)		
*SLC6A4*	Childhood maltreatment	Cognition in psychosis	Aas et al. (91)		
*GRIN2B*	Herpes simplex virus-2 *in utero*	Schizophrenia/psychosis	Demontis et al. (92)		
*CTNNA3*	Cytomegalovirus *in utero*	Schizophrenia/psychosis	Borglum et al. (93)		

**BDNF*, brain-derived neurotrophic factor; *CRHR1*, corticotropine receptor; *HTR2A*, serotonin receptor 2A; *MAOA*, monoamine oxidase A; *NR3C1*, glucocorticoid receptor; *SLC6A4*, serotonin transporter; *COMT*, catechol-*O*-methyl transferase; *AKT1*, serine/threonine-protein kinase; *FKBP5*, glucocorticoid receptor co-chaperone; *GRIN2B*, glutamate NMDA receptor subunit; *CTNNA3*, catenin, cadherin-associated protein*.

The first reported specific gene–environment interaction for a psychotic disorder included a functional polymorphism in the catechol-*O*-methyltransferase (*COMT*) gene. *COMT* codes an enzyme that metabolizes dopamine, the principal neuromediator involved in the positive symptoms of psychosis. A single nucleotide polymorphism (SNP) (rs4680, Val^158^Met) substitutes valine by methionine (Met) at position 158, leading to the production of an enzyme that is much less efficient than the native Val variant. Caspi and colleagues found that use of cannabis in adolescence led to psychotic symptoms and disorders specifically in individuals carrying the more efficient Val alleles at the functional Val^158^Met *COMT* polymorphism (82). While the choice of candidate gene and polymorphism was well justified, the direction of the effect might have been surprising: the more efficient Val allele was associated with sensitivity whilst the less efficient Met allele conferred protection. This finding might have had major implications for personalized prevention of psychosis: a sensitizing genetic variant that explains why many young people remain well even after smoking large amounts of cannabis may help deliver a credible personalized message to those at the highest risk. However, this finding proved difficult to replicate. While initial experimental data supported the interaction (94), several independent studies reported non-replications (83, 95–97) or even findings in the opposite direction (98). It appeared that this gene–environment interaction must have been a false-positive finding. However, recent data suggest that there may be a genuine interaction involving COMT and cannabis. Supportive data have been reported from the genetic and psychosis (GAP) study of first onset psychosis together with an explanation why some previous studies might not have found the expected results: the pathogenic effects of cannabis depends on the proportion of tetrahydrocannabinol and cannabidiol (99, 100). When this is taken into account, the gene–environment interaction as reported by Caspi and colleagues was replicated for adolescent exposure to cannabis with high tetrahydrocannabinol to cannabidiol ratio (101). Another refinement has been reported by taking account of childhood maltreatment in addition to the use of cannabis in adolescence: Alemany and colleagues reported a three-way interaction between the *COMT* genotype Val alleles, childhood maltreatment, and adolescent cannabis use in the etiology of psychotic experiences (84). Most remarkably, this complex three-way interaction was independently replicated by the GROUP investigators: in their sample of Dutch young adults, combination of two *COMT* Val alleles childhood maltreatment and use of cannabis in adolescence was associated with the highest risk of psychotic experiences (85). While the recent refinements are awaiting further tests, the interim conclusion can be made that *COMT* and cannabis are likely to be part of a complex causal mechanism leading to psychotic symptoms and schizophrenia.

In the meantime, another genetic polymorphism has been identified that may moderate the effects of cannabis use in development of psychosis. This started with an investigation of 152 genetic variants in 42 selected candidate genes (86). A polymorphism (rs2494732) in the *AKT1* gene was identified that interacted with the use of cannabis in the pathogenesis of psychosis: carriers of the C/C genotype on rs2494732 were most likely to develop psychotic illness after smoking cannabis. This interaction is not just highly plausible (*AKT1* codes a serine/threonine kinase that relays signal from the cannabinoid receptors), but it appears remarkably robust: the gene–environment interaction between *AKT1* rs2494732 and cannabis replicated across three analyses in the primary report (86). Soon after, an independent replication with the same direction of effect was reported in the GAP study of first-episode psychosis patients and healthy controls (87). This effect was driven by daily use of cannabis increasing the risk of psychosis sevenfold in rs2494732 C allele homozygotes, suggesting that avoidance of heavy use of cannabis is highly advisable for individuals carrying this genotype.

A group of Danish researchers focused on another established environmental risk factor for SMI: exposure to virus infection *in utero* (102–104). In one study, they tested 124 SNPs in five genes encoding components of the NMDA glutamatergic receptor, using 365 cases of schizophrenia and 365 matched healthy controls from the Danish population registry (92). They identified two polymorphisms (rs1805539 and rs1806205) in the *GRIN2B* gene that significantly interacted with maternal positivity for the herpes simplex virus-2 (92). This promising finding is awaiting a replication test.

Additional single candidate gene studies investigated genetic variants with known gene–environment interactions in common mental disorders. The *FKBP5* gene coding a co-chaperone of the glucocorticoid receptor was reported to sensitize individuals to developing post-traumatic stress disorder after being exposed to childhood maltreatment (105). Collip and colleagues found a similar gene–environment interaction involving the same SNPs in *FKBP5* and childhood maltreatment in increasing the risk of experiencing psychotic symptoms in young adults (90). Perhaps the most investigated gene in relationship to environment is *SLC6A4*, which encodes the serotonin transporter. A functional length polymorphism in the promoter of *SLC6A4*, known as 5-hydroxy tryptamine transporter-linked polymorphic region (5-HTTLPR), has been shown to moderate the effects of childhood maltreatment on depression: individuals carrying the short alleles of 5-HTTLPR are prone to develop persistent depressive disorder if they experience maltreatment in childhood (78, 80, 106). Aas and colleagues investigated the interplay between 5-HTTLPR and childhood maltreatment in psychosis and found that the combination of the short 5-HTTLPR alleles and history of childhood maltreatment was associated with cognitive impairment among patients with psychotic disorders (91). This effect was only seen for physical abuse and physical neglect and it did not hold when all types of childhood maltreatment were combined. This finding is waiting for independent replication. A functional polymorphism (Val^66^Met) in the brain-derived neurotrophic factor (*BDNF*) gene has been reported to interact with stressful life events and childhood maltreatment in the development of depression, with Met allele carriers being more likely to develop depression after exposure to adversity (71, 107, 108). From a convenience predominantly student sample, Alemany and colleagues have reported that *BDNF* Met allele carriers with a history of childhood abuse were also more likely to develop psychotic-like experiences (88); this gene–environment interaction has not been replicated in a general population sample of adolescents (89).

Compared to both major depressive disorder and schizophrenia, gene–environment interactions in bipolar disorder have been understudied. Only a single published study has reported that people with bipolar disorder who carried Met alleles at the *BDNF* Val^66^Met polymorphism were more likely to develop depressive episodes following stressful life events than Val allele homozygotes (81).

## Systematic Search for Gene–Environment Interactions

All the studies reviewed above were restricted to the exploration of one or more polymorphisms in one or more genes that were selected based on their presumed functionality in relation to the disorder or the exposure of interest, i.e., they were candidate gene studies. The study of genetic associations across phenotypes has demonstrated that researchers had not been able to select the right candidate genes: most strong genetic associations turned out to be in genes that no one suspected to be involved (109). In addition, most genetic associations reported from candidate gene studies have proven to be false-positive findings perpetuated through publication bias but not replicated in large-scale systematic studies (110). While candidate gene–environment interactions have had better replicability record (Table 2) (69), the fact that study of gene–environment interactions remains largely limited to functional candidate genes is worrying. It is likely that more cases of SMI can be explained by gene–environment interactions involving genetic variants that no one had suspected than those few polymorphisms explored in the above reviewed studies. Therefore, a systematic search for gene–environment interactions across the genome is the essential next step in establishing the etiology of SMI.

To date, only one systematic search for gene–environment interactions in SMI has been carried out. A group of Danish researchers have searched the genome for genetic variants that may sensitize individuals to developing schizophrenia after being exposed to cytomegalovirus *in utero* (93). In 488 cases of schizophrenia and 488 healthy controls from the Danish population registry, they measured antibodies to cytomegalovirus in dried blood spots taken from infants at birth (to carry out the Guthrie test for phenylketonuria) and archived. Since the fetus does not produce its own antibodies, these antibodies are of maternal origin and a proxy of maternal infection with cytomegalovirus during pregnancy. From the same dried blood spots, they also extracted DNA and genotyped over half a million SNPs. They did not test interaction with maternal cytomegalovirus infection for all the genotyped SNPs, because of concerns about statistical power. Instead, they carried out a prioritization step and selected 29,000 polymorphisms that were significantly associated with cytomegalovirus infection in the combined case–control sample. This prioritization was based on a proposal that associations between polymorphisms and an exposure in a case–control sample may be induced by a gene–environment interaction (111) (this reasoning is only applicable to case–control samples, and only makes sense for relatively rare disorders). Among the 29,000 SNPs, the rs7902091 in the *CTNNA3* gene was found to significantly interact with maternal cytomegalovirus infection in causing schizophrenia after correcting for the number of tests performed. It did not reach the accepted genome-wide level of significance. *CTNNA3* encodes a cadherin-associated protein that had been liked to cardiomyopathy, but not suspected to be involved in SMI. It remains to be established whether this gene–environment interaction will prove to be robust in replication.

## Future Outlook for Genes and Environment

Adequately powered genome-wide searches for gene–environment interactions should be a priority for the next decade of research. Since the statistical power for detecting gene–environment interactions is lower than statistical power for detecting direct gene-disorder associations (112, 113), large samples will be needed. Paradoxically, these efforts are held back by the unavailability of reliably assessed environmental exposures rather than genome-wide genotyping. The latest genome-wide analyses of schizophrenia, bipolar disorder, and major depressive disorder involved several tens of thousands of cases and tens of thousands of controls each. Yet the largest investigation of gene–environment interactions in schizophrenia involved fewer than 1000 cases. The situation for bipolar disorder is even more striking, with a near absence of gene–environment studies in spite of substantial shared etiology with both depression and schizophrenia and a large heritability gap left to be explained.

The move to systematic genome-wide gene–environment studies will have to overcome major challenges in addition to sample size (69). Several lines of evidence have shown that the quality of assessment of environmental variables is essential. The replicability of interaction between 5-HTTLPR and childhood maltreatment in leading to persistent depression depends on high-quality assessment of childhood maltreatment with detailed interviews or historically recorded variables (114), the replicability of interactions between COMT and cannabis use may depend on how well the exposure to cannabis is characterized, including age and frequency of use as well as the type of cannabis used (101, 115). In the past, large sample collection studies often discounted on the assessment quality, leading to a negative relationship between study size and quality and non-replications in large samples (116, 117). Therefore, when obtaining environmental variables from large samples, substantial efforts will be required to maintain the quality of assessment of environmental variables.

Another challenge lies in the selection of environmental variables to be assessed. A potentially large number of environmental exposures might be contributing to SMI (Table 1). Yet, with each environmental variable added, the number of potential gene–environment tests increases by the number of genetic variables (which is effectively in the range of 500,000–1,000,000 after taking into account linkage disequilibrium between polymorphisms) and the sample size requirements increase accordingly.

Several initiatives have been launched with the aim to collect a systematic selection of environmental variables in addition to genetic material from moderately large samples (118–120). Obtaining even larger samples would require a degree of coordination between genetic and epidemiological studies. For example, a funding agency may prioritize funding genotyping only for completely assessed samples with high-quality data on environmental exposures or support genetic sample collections in high-quality epidemiological studies of important environmental exposures. Obtaining genetic and environment data from the same rather than separate samples would create significant opportunities without increasing the total cost of research carried out. With some of these initiatives taking place, our understanding of SMI may substantially evolve over the next decade.

## Conflict of Interest Statement

The author declares that the research was conducted in the absence of any commercial or financial relationships that could be construed as a potential conflict of interest. The Guest Associate Editor Helen Fisher declares that, despite having collaborated with the author Rudolf Uher, the review process was handled objectively.
